# No increased risk of short-term complications after radical cystectomy for muscle-invasive bladder cancer among patients treated with preoperative chemotherapy: a nation-wide register-based study

**DOI:** 10.1007/s00345-019-02770-2

**Published:** 2019-04-24

**Authors:** Tomas Jerlström, Ruoqing Chen, Fredrik Liedberg, Ove Andrén, Viveka Ströck, Firas A. S. Aljabery, Abolfazl Hosseini, Amir Sherif, Per-Uno Malmström, Anders Ullén, Truls Gårdmark, Katja Fall

**Affiliations:** 1 Department of Urology, Faculty of Medicine and Health, Örebro University, Örebro, Sweden; 2 Clinical Epidemiology and Biostatistics, School of Medical Sciences, Örebro University, Örebro, Sweden; 3 Clinical Epidemiology Division, Department of Medicine Solna, Karolinska Institutet, 171 76 Stockholm, Sweden; 4 Department of Urology, Skåne University Hospital, Malmö, Sweden; 5 Department of Translational Medicine, Lund University, Malmö, Sweden; 6 Department of Urology, Sahlgrenska University Hospital, Gothenburg, Sweden; 7 Sahlgrenska Academy, Institute of Clinical Sciences, Gothenburg, Sweden; 8 Department of Clinical and Experimental Medicine, Linköping University, Linköping, Sweden; 9 Section of Urology, Department of Molecular Medicine and Surgery, Karolinska Institutet, Stockholm, Sweden; 10 Department of Surgical and Perioperative Sciences, Urology and Andrology, Umeå University, Umeå, Sweden; 11 Department of Urology, Institute of Surgical Sciences, Uppsala, Sweden; 12 PO Bäckencancer, Theme Cancer, Karolinska University Hospital and Department of Oncology-Pathology, Karolinska Institutet, Solna, Sweden; 13 Department of Clinical Sciences, Danderyd Hospital, Karolinska Institutet, Stockholm, Sweden; 14 Department of Medical Epidemiology and Biostatistics, Karolinska Institutet, Stockholm, Sweden

**Keywords:** Bladder cancer, Radical cystectomy, Neoadjuvant chemotherapy, Induction chemotherapy, Postoperative complications

## Abstract

**Purpose:**

Preoperative chemotherapy is underused in conjunction with radical cystectomy (RC) for muscle-invasive bladder cancer (MIBC) due to concerns for complications and delay of surgery. Prospective data on short-term complications from population-based settings with frequent use of preoperative chemotherapy and standardised reporting of complications is lacking.

**Methods:**

We identified 1,340 patients who underwent RC between 2011 and 2015 in Sweden due to MIBC according to the Swedish Cystectomy Register. These individuals were followed through linkages to several national registers. Propensity score adjusted logistic regression was used to calculate odds ratios (ORs) and 95% confidence intervals (CIs) for complications and death within 90 days of surgery, comparing patients receiving preoperative chemotherapy or not.

**Results:**

Minimum two cycles of preoperative chemotherapy were given to 519 (39%) of the patients, who on average tended to be younger, have higher education, better physical status, and more advanced bladder cancer than patients not receiving chemotherapy. After adjusting for these and other parameters, there was no association between treatment with preoperative chemotherapy and short-term complications (OR 1.06 95% CI 0.82–1.39) or mortality (OR 0.75 95% CI 0.36–1.55). We observed a risk reduction for gastrointestinal complications among patients who received preoperative chemotherapy compared with those who did not (OR 0.49 95% CI 0.30–0.81).

**Conclusion:**

This nation-wide population-based observational study does not suggest that preoperative chemotherapy, in a setting with high utilisation of such treatment, is associated with an increased risk of short-term complications in MIBC patients treated with radical cystectomy.

**Electronic supplementary material:**

The online version of this article (10.1007/s00345-019-02770-2) contains supplementary material, which is available to authorized users.

## Introduction

Radical cystectomy (RC) with pelvic lymph node dissection (PLND) and urinary diversion (UD) has been gold standard as treatment of organ-confined muscle-invasive bladder cancer (MIBC) for decades. Since 2008, the European guidelines recommend cisplatin-based neoadjuvant chemotherapy (NAC) prior to surgery if the patient is eligible for such treatment [1, 2]. Most commonly used are either one of the combinations methotrexate, vinblastine, doxorubicin, and cisplatin (MVAC) or gemcitabine and cisplatin (GC) [3]. Preoperative chemotherapy may also be used as induction chemotherapy in patients with more advanced disease [4]. RC is a complex procedure associated with a considerable risk of postoperative morbidity and mortality. The rate of complications ranges from 19 to 92 percent in different studies [5–9], and the wide variation is likely related to whether implementation of standardised reporting guidelines was applied or not [10]. Randomised studies have indicated similar complication rates in patients treated with and without NAC, but have not been designed to assess postoperative complications in a standardised fashion, i.e. using the Clavien–Dindo classification [11–13]. Furthermore, existing observational studies, although generally with retrospective study-designs, based on small numbers of patients and in settings with a low proportion of patients receiving preoperative chemotherapy, have not indicated increased complication risk in association with preoperative chemotherapy [14–18]. Nevertheless, despite a demonstrated survival benefit of 5–8 percent of NAC [11, 19, 20], concerns of a possibly increased risk for complications in the subsequent surgery or delay of the surgery may have contributed to the observed underutilisation [21, 22].

In a setting with high utilisation of preoperative chemotherapy, we have evaluated prospectively collected information on short-term complications after RC in a population-based cohort of MIBC patients treated with or without preoperative chemotherapy.

## Materials and methods

Since 2011, the Swedish National Cystectomy Register prospectively collects data on perioperative parameters and 90-day complications for all RCs performed due to bladder cancer [23]. The coverage of the register during 2011–2015 was 85% according to the Swedish National Board of Health and Welfare [24]. Early complications are assessed according to the Clavien–Dindo classification [25]. We identified all registered patients operated between 2011 and 2015 (*n* = 1918). Using the personal identity number, a unique identifier assigned to all Swedish residents, we linked patient records to the Register of Total Population and Population Changes, and the Longitudinal Integration Database for Health Insurance and Labor Market Studies. The latter provided information on attained educational level as an indicator of socioeconomic status.

We excluded patients with an ambiguous personal identity number (*n* = 2), clinically non-muscle invasive disease (cT < T2) (*n* = 559), with missing information on clinical stage (cTx) (*n* = 2), preoperative chemotherapy treatment (*n* = 7), or on all complication and reoperation variables (*n* = 8). Thus, 1340 patients diagnosed with clinically muscle-invasive disease (cT2-4) treated with cystectomy remained for the analyses.

### Exposure and outcomes assessment

The exposure of interest was defined as registered use of two or more cycles of preoperative chemotherapy. During the study period the register did not further specify the exact number of chemotherapy courses or type of chemotherapy. Primary outcome was short-term complications (occurring within 90 days of surgery) including death. All patients were followed until the 90th day after surgery.

### Covariates

Baseline clinical information included patient’s sex, age, body mass index (BMI), educational level, American Society of Anesthesiology (ASA) physical status score, previous pelvic surgery or radiation, and clinical tumor stage and nodal status (cT, cN). Perioperative parameters included operation time, estimated blood loss, transfusion, mode of operation (open or robotic assisted), type of urinary diversion, pelvic lymph node dissection template, and length of stay. Hospital volume was defined as mean annual number of operations performed per hospital during the study period. Highest attained educational level was used as an indicator of socioeconomic status and stratified as compulsory school, upper secondary school, and university level.

### Statistical analyses

Continuous variables are presented as either mean with standard deviation or medians with interquartile ranges (IQR), and categorical variables as frequencies with percentages. Differences between the two study groups were evaluated using T-test or Wilcoxon rank-sum test for continuous variables, and Pearson’s Chi square test or Fisher’s exact test for categorical variables. Logistic regression analyses were conducted, yielding odds ratios (ORs) with 95% confidence intervals (CIs) for binary outcome variables. Multinomial logistic regression was performed for categorical outcome variable (highest Clavien–Dindo score), where no complication was used as the reference category. We adjusted for a propensity score which was computed by modelling a logistic regression with preoperative chemotherapy as the dependent variable and age at surgery, sex, BMI, operation time, estimated blood loss, type of urinary diversion, previous surgery or radiation therapy in the small pelvis, educational level, cT-stage, ASA-score, and hospital volume as the independent variables. *P* values less than 0.05 were considered to be statistically significant.

In a sensitivity analysis, patients with cT4b and/or cN + -tumors were excluded to examine the potential influence of induction chemotherapy.

Data were analysed using SAS (version 9.4; SAS Institute Inc., Cary, NC).

## Results

Of the 1340 patients with MIBC, 519 (39%) received two or more cycles of preoperative chemotherapy. The majority of the exposed and unexposed groups were men (75% and 73%, respectively). Patients receiving preoperative chemotherapy had a lower mean age, higher level of education, lower ASA-score, higher proportion of undergoing previous pelvic surgery or radiation, and more advanced tumors than patients not receiving chemotherapy (Table 1). Among the 802 patients (60% of the study population) operated in academic centers, 372 (46%) received preoperative chemotherapy. In the remaining 519 patients operated in non-academic centers, the corresponding figure is 136 (26%).

**Table 1:** Baseline clinical characteristics of patients with MIBC treated with cystectomy and urinary diversion

Characteristics	Total (*N* = 1340)	No preoperative chemotherapy (*N* = 821)	Preoperative chemotherapy (*N* = 519)	*P* value
Sex (*N* [%])^a^				
Male	990 (73.9)	600 (73.1)	390 (75.1)	0.40
Female	350 (26.1)	221 (26.9)	129 (24.9)	
Age (mean [SD])^b^	70.84 (8.4)	73.91 (7.7)	65.97 (7.3)	< 0.001
BMI (*N* [%])^a^				
< 18.5	28 (2.1)	18 (2.2)	10 (1.9)	0.33
18.5–24.9	568 (42.4)	361 (44.0)	207 (39.9)	
25–29.9	516 (38.5)	312 (38.0)	204 (39.3)	
≥ 30	211 (15.8)	118 (14.4)	93 (17.9)	
Missing	17 (1.3)	12 (1.5)	5 (1.0)	
Education (*N* [%])^a^				
Compulsory	500 (37.3)	336 (40.9)	164 (31.6)	0.003
Upper secondary	556 (41.5)	320 (39.0)	236 (45.5)	
University level	275 (20.5)	158 (19.2)	117 (22.5)	
Missing	9 (0.7)	7 (0.8)	2 (0.4)	
ASA-classification (*N* [%])^a^				
1	161 (12.0)	89 (10.8)	72 (13.9)	0.02
2	727 (54.2)	431 (52.5)	296 (57.0)	
3	424 (31.6)	280 (34.1)	144 (27.8)	
4	18 (1.3)	15 (1.8)	3 (0.6)	
Missing	10 (0.8)	6 (0.7)	4 (0.8)	
Previous pelvic surgery or radiation (*N* [%])^a^				
No	1109 (82.8)	660 (80.4)	449 (86.5)	0.01
Yes	219 (16.3)	154 (18.8)	65 (12.5)	
Missing	12 (0.9)	7 (0.8)	5 (1.0)	
Staging (*N* [%])				
cT category^a^				
T2	987 (73.7)	627 (76.4)	360 (69.4)	0.03
T3	249 (18.6)	139 (16.9)	110 (21.2)	
T4a	87 (6.5)	45 (5.5)	42 (8.1)	
T4b	17 (1.3)	10 (1.2)	7 (1.4)	
cN category^a^				
N0	1122 (83.7)	714 (87.0)	408 (78.6)	<0.001
N + (incl.N1-3, N +)	146 (10.9)	61 (7.4)	85 (16.4)	
Missing (incl.Nx)	72 (5.4)	46 (5.6)	26 (5.0)	
Hospital volume (no. of operations/year)^a^				
≤ 20	404 (30.2)	275 (33.5)	129 (24.9)	<0.001
20–50	428 (31.9)	220 (26.8)	208 (40.1)	
> 50	507 (37.8)	326 (39.7)	181 (34.9)	
Missing	1 (0.1)	0 (0.0)	1 (0.2)	

^a^Chi square test or Fisher’s exact test

^b^*T* test

Perioperative parameters in the two groups are presented in Table 2. Patients treated with preoperative chemotherapy had longer operating time, received blood transfusions more frequently (33% vs 24%), even if the estimated median blood loss was lower (600 vs 700 ml), more frequently had robotic surgery (29% vs 20%), were more likely to have an orthotopic neobladder substitution (16% vs 5%), and more frequently had a pelvic lymph node dissection extended up to the aortic bifurcation (28% vs 14%).

**Table 2:** Perioperative parameters of patients with MIBC treated with cystectomy and urinary diversion

Characteristics	Total (*N* = 1340)	No preoperative chemotherapy (*N* = 821)	Preoperative chemotherapy (*N* = 519)	*P* value
Operation time (minutes, mean [SD])^a^	332 (114)	313 (108)	360 (117)	< 0.001
Estimated blood loss (median [IQR])^b^	650 (300, 1300)	700 (300, 1400)	600 (250, 1100)	< 0.001
Transfusion (*N* [%])^c^				
No	972 (72.5)	625 (76.1)	347 (66.9)	< 0.001
Yes	368 (27.5)	196 (23.9)	172 (33.1)	
Median (units) (IQR)^b^	2 (2, 4)	2.5 (2, 5)	2 (2, 4)	0.49
Mode of operation (*N* [%])^c^				
Open	1024 (76.4)	653 (79.5)	371 (71.5)	< 0.001
Robotic	315 (23.5)	167 (20.3)	148 (28.5)	
Missing	1 (0.1)	1 (0.1)	0 (0.0)	
Diversion (*N* [%])^c^				
Ileal conduit	1179 (88.0)	749 (91.2)	430 (82.9)	< 0.001
Neobladder	124 (9.3)	42 (5.1)	82 (15.8)	
Continent cutaneous	10 (0.8)	3 (0.4)	7 (1.4)	
Other	26 (1.9)	26 (3.2)	0 (0.0)	
Missing	1 (0.1)	1 (0.1)	0 (0.0)	
Pelvic lymph node dissection (*N* [%])^c^				
Aortic bifurcation	264 (19.7)	117 (14.2)	147 (28.3)	< 0.001
Iliac bifurcation	782 (58.4)	479 (58.3)	303 (58.4)	
Obturator fossae	107 (8.0)	73 (8.9)	34 (6.6)	
Enlarged only	36 (2.7)	27 (3.3)	9 (1.7)	
None	149 (11.1)	124 (15.1)	25 (4.8)	
Missing	2 (0.1)	1 (0.1)	1 (0.2)	
Length of stay (median [IQR])^b^	13 (10, 17)	13 (10, 18.5)	13 (10, 16)	0.003

^a^*T* test

^b^Wilcoxon rank-sum test

^c^Chi square test or Fisher’s exact test

The occurrence of short-term complications was observed in 49% and 47% of patients treated with or without chemotherapy, respectively. The chemotherapy group had a higher proportion of Clavien–Dindo grade I–II complications (24% vs 20%) and a lower proportion of Clavien–Dindo grade III–V complications (22% vs 23%) than the non-chemotherapy group. No differences between the groups were, however, observed in the propensity-adjusted multivariable models overall (OR 1.06 95% CI 0.82–1.39), or for complications of each separate Clavien–Dindo grade (Table 3). When assessing different types of complications, the proportion of overall gastrointestinal complications was found to be lower in the chemotherapy group (7%) than in the non-chemotherapy group (11%), rendering a propensity score adjusted OR of 0.49 (95% CI 0.30–0.81). Further analyses within this group indicated mechanical ileus as the underlying explanation (adjusted OR 0.47, 95% CI 0.27–0.83). Overall, small proportions of patients in both groups experienced specific complications (Supplementary Table 1), but the power to assess differences between subgroups in adjusted models is limited. A lower proportion of reoperations for mechanical ileus was suggested in the chemotherapy group (1.3% vs 3.2%) (*P* = 0.10), however, as well as a lower proportion of postoperative pneumonia (0.4% vs 4.1%) (*P* < 0.001). No other clear differences in complications were discernable between the groups. In a sensitivity analysis, after excluding patients with cT4b and/or cN + (who potentially received induction chemotherapy), the overall association between preoperative chemotherapy and short-term complications was essentially unchanged (OR = 1.16 95% CI = 0.87–1.54).

**Table 3:** Risk for complications, reoperations and death within 90 days from cystectomy comparing patients with and without preoperative chemotherapy treatment

Outcomes	Total (*N* = 1340)	No preoperative chemotherapy (*N* = 821)		Preoperative chemotherapy (*N* = 519)	
	*N* (%)	*N* (%)	OR (95%C)	*N* (%)	OR (95%CI)^b^
Any complication	636 (47.5)	382 (46.5)	Ref	254 (48.9)	1.06 (0.82–1.39)
Highest Clavien–Dindo^a^					
I–II	291 (21.7)	165 (20.1)	Ref	126 (24.3)	1.18 (0.84–1.65)^c^
III	238 (17.8)	140 (17.1)	Ref	98 (18.9)	1.12 (0.78–1.61)^c^
IV	39 (2.9)	26 (3.2)	Ref	13 (2.5)	0.70 (0.31–1.57)^c^
V	25 (1.9)	21 (2.6)	Ref	4 (0.8)	0.59 (0.14–2.40)^c^
Gastrointestinal complication	128 (9.6)	94 (11.4)	Ref	34 (6.6)	0.49 (0.30–0.81)
Cardiovascular complication	74 (5.5)	47 (5.7)	Ref	27 (5.2)	0.96 (0.53–1.74)
Infectious complication	338 (25.2)	197 (24.0)	Ref	141 (27.2)	1.07 (0.79–1.45)
Abdominal wall/stoma complication	153 (11.4)	93 (11.3)	Ref	60 (11.6)	1.03 (0.68–1.57)
Urinary tract complication	102 (7.6)	61 (7.4)	Ref	41 (7.9)	1.15 (0.70–1.89)
Nerve damage	7 (0.5)	5 (0.6)	Ref	2 (0.4)	0.26 (0.05–1.51)
Unscheduled readmission	347 (25.9)	208 (25.3)	Ref	139 (26.8)	1.21 (0.89–1.64)
Reoperation	173 (12.9)	106 (12.9)	Ref	67 (12.9)	1.02 (0.68–1.51)
Death	66 (4.9)	52 (6.3)	Ref	14 (2.7)	0.75 (0.36–1.55)

^a^Numbers do not add to total due to missing data

^b^Model with propensity score

^c^Relative risk ratio was estimated using multinomial logistic regression using patients without complication as the comparison group

## Discussion

In this nation-wide population-based study, representing a real-world setting with routine health care, preoperative chemotherapy in conjunction with radical cystectomy due to MIBC was not associated with overall risk of developing complications within 90 days of surgery. These findings are generally in line with previous retrospective observational studies [14–18]. Among the available randomised studies investigating NAC, three report postoperative complications, albeit without applying standardised complication reporting, i.e. using the Clavien–Dindo classification. Grossman et al. observed similar proportions (13%) of high-grade complication in the NAC and the non-NAC groups during the postoperative period, without further defining the time point for when the assessment was performed [11]. The study by Kitamura et al. specifies surgery-related complication as a secondary endpoint, and reports a higher rate of anastomotic leaks (12% vs 2%) and a lower rate of lymph leakage (2% vs 12%) in the NAC group, although the sample size was small (*n* = 130) [13]. In the larger randomised BA06 30894-trial, including 488 patients receiving RC with or without NAC, no increased risk of complications was reported in the NAC arm [14]. In line with our data, however, the pioneering observational report from Memorial Sloan Kettering Cancer Center, suggests, although not statistically significant, that preoperative chemotherapy is associated with a lower risk of developing high-grade complications (OR 0.51 95% CI 0.25–1.01) [7]. Hence, one might speculate that patients selected for preoperative chemotherapy have a lower baseline risk for developing complications compared with patients not selected for chemotherapy, although the results were similar in the present study. The 90-day mortality in patients with preoperative chemotherapy in the present series is low and comparable to previously published series without chemotherapy (5.2%) [26]. Blood loss was furthermore comparable to that observed in a large historical series where only a minority of 12% received preoperative chemotherapy [10].

We further observed a lower risk of gastrointestinal complications in the chemotherapy group, primarily related to a reduced incidence of mechanical ileus. This may be at least partially explained by a higher rate of orthotopic neobladder substitution (ONS) in the chemotherapy group, as it has previously been described that patients with ONS have reduced risks of hospitalisation due to ileus at long-term follow-up [27]. Our finding of increased use of transfusions in the chemotherapy group despite lower blood loss could be related to bone marrow depression and lower haemoglobin levels prior to surgery, this could not be confirmed as preoperative haemoglobin levels were not available through the register, but has been previously described [28]. The difference in the risk for pneumonia found between the chemotherapy and non-chemotherapy groups (0.4%vs 4%) could potentially be explained by the lower occurrence of gastrointestinal complications, especially ileus, since use of nasogastric tube, which was probably used more often among the patients with ileus, is a known risk factor for pneumonia [29].

Chemotherapy was more often used in academic centers and centers with larger surgical volume, hence having a better adherence to guidelines, compared to non-academic centers and centers with lower volume. Further subgroup analysis was not done since other factors than preoperative chemotherapy most likely could affect the rate of complications. Such factors might be the volume itself or more patients receiving orthotopic neobladder substitution, with more severe comorbidity or advanced tumor stage, that are expected to be referred to academic and high-volume centers.

The strengths of this study include the use of prospectively collected data from the nation-wide population-based cystectomy register with a high coverage and linkages with essentially complete population-based registers. Compared with previous observational studies [14–18] performed in settings with lower utilisation of preoperative chemotherapy and with retrospectively collected information, all but one with a smaller sample size, the present study thus presents some advantages. However, some limitations should also be noted. A major potential caveat is related to the observational study design and the inherent selection of patients into the two treatment groups; treatment selection for preoperative chemotherapy may be correlated to factors associated with a lower risk of complications such as younger age and less comorbidity. Although propensity-score adjustments for these and other perioperative factors were made with limited impact on risk estimates, influence of unmeasured or unknown confounders cannot be excluded. Reasons for residual confounding may for example include contraindications for cisplatin-based combination chemotherapy, such as decreased renal function, impaired performance status, hearing loss and comorbidity including ischemic heart disease. Another limitation is that although this currently constitutes the largest study in terms of patients treated with preoperative chemotherapy, the statistical power to rule out uncommon complications is limited.

The number of cycles of chemotherapy was not registered, patients receiving two or more cycles of chemotherapy were registered in a yes/no fashion. Patients who only received one treatment cycle of chemotherapy or developed complications from chemotherapy preventing surgery, could further not be identified in the register. The results may thus not be generalisable to all patients considered eligible for chemotherapy. Due to recent developments of the register, these patients will, however, be possible to identify in future studies, as well as the number and type of chemotherapy courses given in a pure neoadjuvant setting and an induction setting, respectively. During the study period registration of treatment did not distinguish between NAC and induction chemotherapy. In the former case, at least two cycles of cisplatin-based chemotherapy were required to be classified as NAC. The latter group of patients received, if eligible, cisplatin-based chemotherapy, but also possibly other types of chemotherapy such as carboplatin-based combinations. Although the number of treatment cycles in induction therapy may exceed that of NAC [30], the treatments may be comparable for the purpose of assessing complications. Supporting that, exclusion of patients most likely to have received induction chemotherapy (cT4b and/or cN + stage tumors) in this study did not change the overall results.

In conclusion, these data suggest that preoperative chemotherapy in a setting with high utilisation of preoperative chemotherapy, is not associated with an increased risk of short-term complications in MIBC patients undergoing radical cystectomy.

## Electronic supplementary material

Below is the link to the electronic supplementary material.

Supplementary material 1 (DOCX 22 kb)
