# Supplementary material for: No increased risk of short-term complications after radical cystectomy for muscle-invasive bladder cancer among patients treated with preoperative chemotherapy: a nation-wide register-based study
Source: World J Urol. 2019 Apr 24;38(2):381–8. doi: 10.1007/s00345-019-02770-2 (PMC6994427; doi:10.1007/s00345-019-02770-2)
Supplement: Supplementary file 1 — Supplementary material 1 (DOCX 22 kb) [file 345_2019_2770_MOESM1_ESM.docx]

| **Supplementary Table 1. Complications, reoperations and death within 90 days from cystectomy comparing patients with and without preoperative chemotherapy treatment** | | | | | | |
| --- | --- | --- | --- | --- | --- | --- |
| **Outcomes** | **Total (N=1340)** |  | **No preoperative chemotherapy (N=821)** |  | **Preoperative chemotherapy (N=519)** | P value^a^ |
|  | **N (%)** |  | **N (%)** |  | **N (%)** |  |
| **Any complication** | 636 (47.5) |  | 382 (46.5) |  | 254 (48.9) | 0.59 |
| Highest Clavien-Dindo^b^ |  |  |  |  |  |  |
| I-II | 291 (21.7) |  | 165 (20.1) |  | 126 (24.3) | 0.31^c^ |
| III-V | 302 (22.5) |  | 187 (22.8) |  | 115 (22.2) |  |
|  |  |  |  |  |  |  |
| **Any complication** | 636 (47.5) |  | 382 (46.5) |  | 254 (48.9) | 0.59 |
| Highest Clavien-Dindo^b^ |  |  |  |  |  |  |
| I-IV | 568 (42.4) |  | 331 (40.3) |  | 237 (45.7) | 0.04^d^ |
| V | 25 (1.9) |  | 21 (2.6) |  | 4 (0.8) |  |
|  |  |  |  |  |  |  |
| **Death** | 66 (4.9) |  | 52 (6.3) |  | 14 (2.7) | 0.003 |
| **Cause of Death** |  |  |  |  |  |  |
| Bladder cancer | 27 (2.0) |  | 20 (2.4) |  | 7 (1.4) | 0.01 |
| Other | 32 (2.4) |  | 27 (3.3) |  | 5 (1.0) |  |
| Unknown | 7 (0.5) |  | 5 (0.6) |  | 2 (0.4) |  |
|  |  |  |  |  |  |  |
| **Gastrointestinal complication** | 128 (9.6) |  | 94 (11.5) |  | 34 (6.6) | 0.005 |
| ***Mechanical ileus*** | 89 (6.6) |  | 66 (8.0) |  | 23 (4.4) | 0.02 |
| Reoperation | 33 (2.5) |  | 26 (3.2) |  | 7 (1.3) | 0.10 |
| ***Insufficency of enteric anastomosis*** | 20 (1.5) |  | 13 (1.6) |  | 7 (1.3) | 0.81 |
| Reoperation | 20 (1.5) |  | 13 (1.6) |  | 7 (1.3) | 0.81 |
| ***Bowel fistula*** | 25 (1.9) |  | 19 (2.3) |  | 6 (1.2) | 0.22 |
| Reoperation | 15 (1.1) |  | 10 (1.2) |  | 5 (1.0) | 0.79 |
|  |  |  |  |  |  |  |
| **Cardiovascular complication** | 74 (5.5) |  | 47 (5.7) |  | 27 (5.2) | 0.77 |
| ***Deep venous thrombosis*** | 24 (1.8) |  | 13 (1.6) |  | 11 (2.1) | 0.63 |
| ***Pulmonary embolism*** | 25 (1.9) |  | 14 (1.7) |  | 11 (2.1) | 0.68 |
| ***Deep venous thrombosis and/or pulmonary embolism*** | 46 (3.4) |  | 25 (3.0) |  | 21 (4.0) | 0.53 |
| ***Myocardial infarction*** | 11 (0.8) |  | 9 (1.1) |  | 2 (0.4) | 0.39 |
| ***Stroke*** | 5 (0.4) |  | 5 (0.6) |  | 0 (0.0) | 0.15 |
| ***Periferal artery thrombosis*** | 8 (0.6) |  | 5 (0.6) |  | 3 (0.6) | 0.90 |
|  |  |  |  |  |  |  |
| **Infectious complication** | 338 (25.2) |  | 197 (24.0) |  | 141 (27.2) | 0.42 |
| ***Sepsis*** | 134 (10.0) |  | 76 (9.6) |  | 55 (10.6) | 0.68 |
| ***Pneumonia*** | 36 (2.7) |  | 34 (4.1) |  | 2 (0.4) | <0.001 |
| ***Pyelonefritis*** | 40 (3.0) |  | 19 (2.3) |  | 21 (4.0) | 0.12 |
| ***Lymfocele*** | 41 (3.1) |  | 23 (2.8) |  | 18 (3.5) | 0.63 |
| ***Other infections*** | 130 (9.7) |  | 70 (8.5) |  | 60 (11.6) | 0.12 |
|  |  |  |  |  |  |  |
| **Abdominal wall/stoma complication** | 153 (11.4) |  | 93 (11.3) |  | 60 (11.6) | 0.88 |
| ***Wound infection*** | 69 (5.1) |  | 42 (5.1) |  | 27 (5.2) | 0.92 |
| Reoperation | 10 (0.7) |  | 8 (1.0) |  | 2 (0.4) | 0.49 |
| **Wound dehiscence** | 72 (5.4) |  | 44 (5.4) |  | 28 (5.4) | 0.90 |
| Reoperation | 63 (4.7) |  | 37 (4.5) |  | 26 (5.0) | 0.53 |
| ***Incisional hernia*** | 11 (0.8) |  | 7 (0.9) |  | 4 (0.8) | 0.91 |
| Reoperation | 4 (0.3) |  | 3 (0.4) |  | 1 (0.2) | 0.87 |
| ***Parastomal hernia*** | 0 (0.0) |  | 0 (0.0) |  | 0 (0.0) | N/A |
| ***Necrosis of stoma*** | 7 (0.5) |  | 3 (0.4) |  | 4 (0.8) | 0.55 |
| Reoperation | 3 (0.2) |  | 2 (0.2) |  | 1 (0.2) | 0.73 |
| ***Prolaps of stoma*** | 0 (0.0) |  | 0 (0.0) |  | 0 (0.0) | N/A |
| ***Other abdominal wall/stoma complication*** | 7 (0.5) |  | 6 (0.7) |  | 1 (0.2) | 0.43 |
| Reoperation | 3 (0.2) |  | 3 (0.4) |  | 0 (0.0) | 0.42 |
|  |  |  |  |  |  |  |
| **Urinary tract complication** | 102 (7.6) |  | 61 (7.4) |  | 41 (7.9) | 0.80 |
| ***Stricture of ureteroenteric anastomis*** | 33 (2.5) |  | 22 (2.7) |  | 11 (2.1) | 0.67 |
| Reoperation | 12 (0.9) |  | 9 (1.1) |  | 3 (0.6) | 0.52 |
| ***Stricture of pouch outlet*** | 1 (0.1) |  | 1 (0.1) |  | 0 (0.0) | 0.78 |
| Reoperation | 1 (0.1) |  | 1 (0.1) |  | 0 (0.0) | 0.78 |
| ***Leakage of ureteroenteric anastomosis*** | 44 (3.3) |  | 24 (2.9) |  | 20 (3.9) | 0.52 |
| Reoperation | 14 (1.0) |  | 6 (0.7) |  | 8 (1.5) | 0.36 |
| ***Stricture of urethral anastomosis*** | 2 (1.5) |  | 1 (0.1) |  | 1 (0.2) | 0.83 |
| Reoperation | 0 (0.0) |  | 0 (0.0) |  | 0 (0.0) | N/A |
| ***Other complications of urinary tract*** | 20 (1.5) |  | 13 (1.6) |  | 7 (1.3) | 0.81 |
| Reoperation | 5 (0.4) |  | 3 (0.4) |  | 2 (0.4) | 1.00 |
| ^a^ *P* values from Chi-Square test or Fisher’s Exact test which are used to compare the distribution of outcomes (no, yes, and missing) between two groups. | | | | | | |
| ^b^ Numbers do not add to total due to missing data | | | | | | |
| ^c^ Comparison based on the distribution of complication (no, yes [highest Clavien-Dindo I-II, III-V, missing], missing) | | | | | | |
| ^d^ Comparison based on the distribution of complication (no, yes [highest Clavien-Dindo I-IV, V, missing], missing) | | | | | | |
